# Management of Wilson disease across Europe: an international physician-oriented survey by the ERN-RARE Liver group

**DOI:** 10.1186/s13023-025-04103-6

**Published:** 2025-11-11

**Authors:** Frederik Teicher Kirk, Karina Stubkjær Rewitz, Zoe Mariño, Eduardo Couchonnal, Nicolas Lanthier, Wiebke Papenthin, Marina Berenguer, Aurelia Poujois, Dominique Debray, Aftab Ala, Luis García-Villarreal, Tudor Lucian Pop, Gerald Denk, Piotr Socha, Thomas Damgaard Sandahl

**Affiliations:** 1 European Reference Network on Rare Liver Disorders (ERN-RARE Liver), Hamburg, Germany; 2 Department of Hepatology and Gastroenterology, Aarhus University Hospital, Palle Juul-Jensens Blvd. 99, Aarhus, 8200 Denmark; 3 Liver Unit, Hospital Clínic, IDIBAPS, CIBERehd, Universitat de Barcelona, Barcelona, Spain; 4 Unité d’Hépatologie, gastroentérologie et Nutrition pédiatrique, Hôpital Femme Mère et Enfant, Hospices Civils de Lyon, Bron, France; 5 Service d’Hépato-Gastroentérologie, Cliniques universitaires Saint-Luc, Brussels, Belgium; 6 German Association for Wilson Disease Patients (Morbus Wilson e.V.), Kürnach, Germany; 7 Hepatology and Liver Transplant Unit, IIS La Fe & CIBER-EHD, Universitary and Politecnic Hospital La Fe, Valencia, Spain; 8 European Reference Network for Hereditary Metabolic Disorders (MetabERN), Udine, Italy; 9 Centre de Référence de la Maladie de Wilson et autres Maladies Rares Liées au Cuivre, Paris, France; 10 Institute of Liver Studies, King’s College Hospital, London, UK; 11 Digestive Diseases Service, Complejo Hospitalario Universitario Insular Materno Infantil (CHUIMI) and Instituto Universitario de Investigaciones Biomédicas y Sanitarias (IUIBS), Universidad Las Palmas Gran Canaria, Las Palmas de Gran Canaria, Spain; 12 Second Pediatric Discipline, “Iuliu Hatieganu” University of Medicine and Pharmacy Cluj-Napoca, Center of Expertise in Pediatric Liver Rare Disorders, Emergency Clinical Hospital for Children Cluj-Napoca, Cluj-Napoca, Romania; 13 Department of Medicine II, University Hospital, LMU Munich, Munich, Germany; 14 Department of Gastroenterology, Hepatology, Nutritional Disorders and Pediatrics, Children’s Memorial Health Institute, Warsaw, Poland

**Keywords:** Health equality, Variation in care, Medical management, Rare disease, Disease, European reference network

## Abstract

**Background:**

Wilson disease (WD) is a rare disorder resulting in copper overload. Diagnosis and treatment are complex and highly specialized. We aimed to investigate the management of WD across Europe in line with the mission and framework of the European Reference Network on Rare Liver Disease (ERN-RARE Liver).

**Methods:**

A 37-item questionnaire was distributed among European WD centers. Questions related to WD included diagnosis, treatment, monitoring, patient perspectives, and background information. Responding centers were classified as small or large by the number of patients seen per year (</≥ 30/year).

**Results:**

Sixty-two physicians from 20 countries responded. 58 were included in the analysis. Most physicians were hepatologists. A high, but incomplete degree of adherence to the international guidelines and Leipzig criteria was found. The majority of centers had a wide range of diagnostic tools available, with the larger being more likely to offer a broader range of standard and research-led diagnostic tools. Although different WD medications were widely available, 8 (21%) of the small centers did not offer trientine, in 4 cases, due to cost. Several areas with variations in responses were also demonstrated, notably in recommendations of low copper diets, initial recognition and management of neurologic WD patients, and degree of patient organization collaboration.

**Conclusions:**

Overall, we found uniformity in the management of WD across European WD centers. Nevertheless, variations in key areas were identified, reflecting a lack of robust evidence, thus providing a guide for future research.

**Supplementary Information:**

The online version contains supplementary material available at 10.1186/s13023-025-04103-6.

## Background

Wilson Disease (WD) is a rare genetic disorder characterized by variants of the ATP7B gene and impaired copper metabolism [1]. Consequently, patients with WD experience copper accumulation in the liver and brain, leading to different phenotypical presentations and, if untreated, death [2]. WD affects approximately 1:30.000 people [3, 4]. Diagnosis of WD is complex and may be aided by the use of the Leipzig scoring system based on, e.g., clinical, biochemical, histological, and genetic data [5].

Several therapeutic options exist. In Europe, trientine (TRI), D-penicillamine (PEN), and zinc (Zn) are available [2, 6]. These compounds exert their action either by increasing urinary excretion, decreasing intestinal copper uptake, or a combination of the two [7–9]. Adherence is essential for patients with WD as normal life expectancy can be achieved [2, 10, 11]. However, monitoring treatment effects in WD is challenging and is based on several tools, including calculation of non-ceruloplasmin bound copper (cNCC), direct measurement of non-ceruloplasmin bound copper (CuEXC), and 24-hour urinary copper excretion (24 H-UCE) [12–14].

Multiple well-established international guidelines on the management of WD exist [1, 2], and several local guidelines have been established. Due to the rarity and complexity of WD, strong scientific evidence for many important aspects of WD management is lacking – leading to some variability in existing guidelines.

The European Reference Network on Rare Liver Disorders (ERN-RARE Liver) consists of both physicians and patient advocates. Its purpose is to improve clinical knowledge and patient care in rare liver diseases across Europe, through close collaboration between healthcare facilities, physicians, and patient organizations. Ultimately, promoting equality to high levels of medical care for patients with rare diseases in Europe. This is achieved through the development of guidelines, international clinical collaborations, standardization of care, patient education, and public involvement and engagement.

The aim of this study was (i) to investigate the management and care of WD, including adherence to guidelines for WD, across European centers, (ii) to explore areas of patient care in which stronger evidence is required. To achieve this, a physician-oriented survey was developed in an international collaboration through ERN-RARE Liver.

## Methods

### Study design

This cross-sectional online survey-based study was designed to investigate current clinical practice in the diagnosis and management of WD across European WD centers.

The survey was developed through an iterative process within the ERN-RARE Liver Wilson working group, involving both physicians and a patient advocate.

The survey was distributed by the ERN RARE-Liver working group and hosted by the European Union Survey site https://ec.europa.eu/eusurvey/.

### Respondents

Respondents were clinicians involved with WD management through their employment at European medical centers. As there was no exhaustive list of all European medical centers involved with WD management, the survey was initially distributed to relevant physicians who were registered with the ERN Wilson working group or who were personally known by members of the working group. We then requested that initial recipients distribute the list to relevant physicians in their own country. Responses from centers that did not treat WD were excluded and were not analyzed. If more than one reply was given from the same department, only the first reply was included in the analysis.

Physicians from the original distribution list who had not replied were contacted by mail one and two months after receiving the survey to increase response rates. The survey was live for five months, and no compensation was given for completing the survey. 

Participants consented through their response to the survey, and all responses were anonymized. Centers were divided into small and large centers based on the number of patients seen each year. Centers seeing < 30 patients were defined as ‘small’, while centers seeing more than ≥ 30 patients per year were defined as ‘large’.

### Survey

This internet-based survey consisted of thirty-seven separate questions divided into four sections. The first section (ten questions) provided information about the responding physician and the medical center, such as specialization, country of practice, number of patients seen yearly, and number of new patients yearly.

The second section (eight questions) focused on WD diagnosis. Questions included adherence to guidelines, diagnostic tools, initial symptoms, and use of specialist consults.

The third section (fourteen questions) provided information on treatment and monitoring of WD, with questions on choice of initial treatment, maintenance treatment, and treatment availability.

The fourth section (five questions) related to frequency of follow-up, family screening choices, and engagement with patient organizations. The full list of questions is shown in Supplementary Materials S1.

The survey was distributed prior to the publication of the AASLD and EASL guideline updates on WD Management [1, 2], as such, survey questions on guideline adherence related to the 2008 AASLD and 2012 EASL versions [15, 16].

### Statistical analysis

Responses were imported to STATA version 18.0 (StataCorp LP, College Station, TX). Descriptive statistical analysis was performed due to the nature of the data. Data was subsequently stratified to small and large centers, and normality of data was tested using histograms and QQ plots.

Data is presented as (n, %) unless otherwise specified.

Graphpad Prism version 10.0.0 (Graphpad Software, San Diego, CA) was used to generate figures.

## Results

**Fig. 1:**
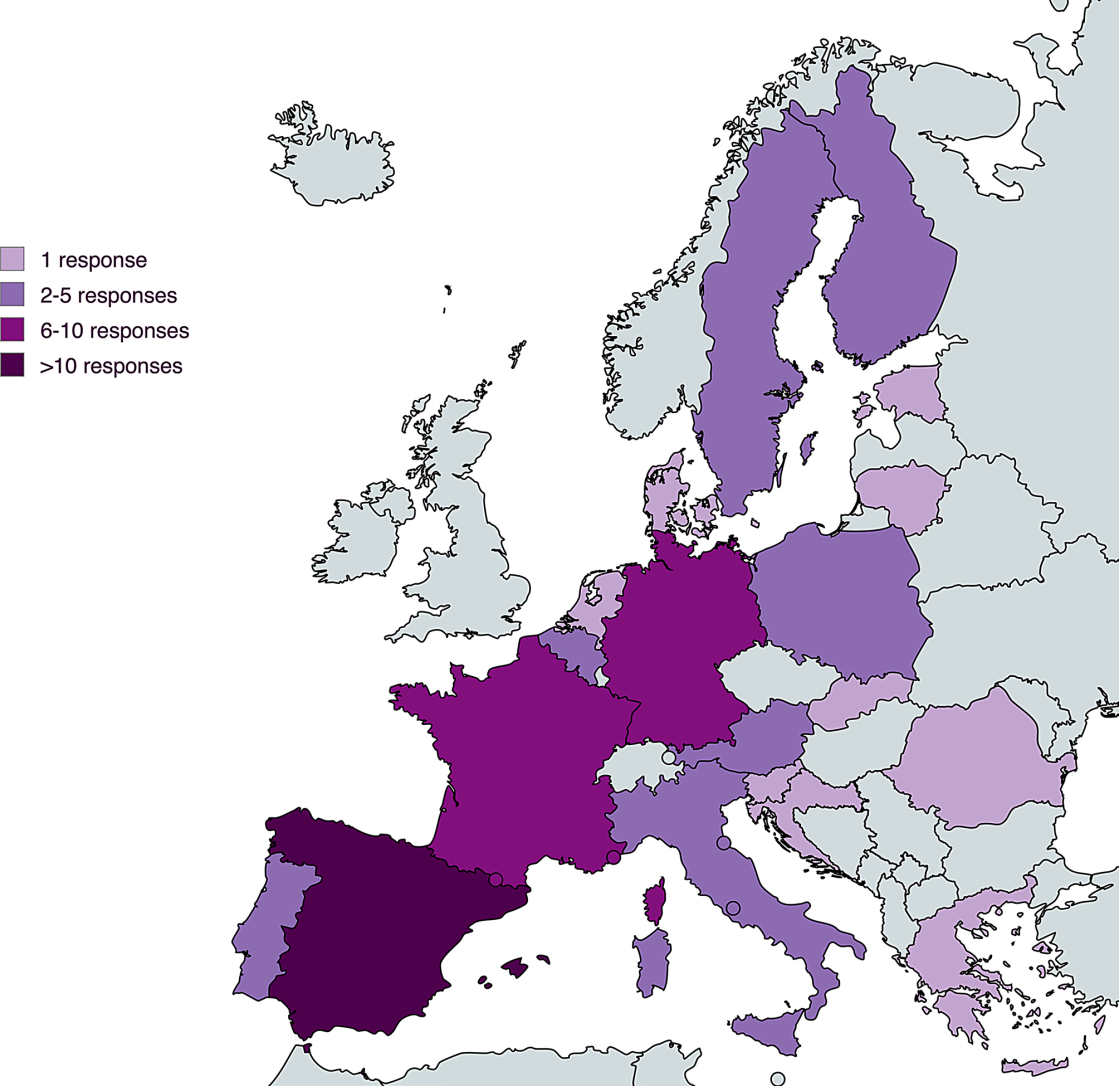
Responses per country. Developed using mapchart.net under a creative commons license

The survey was distributed on September 30th, 2022, and responses were collected until March 2nd, 2023. Sixty-two responses were collected, and four responses were excluded because of multiple responses from the same person (*n* = 2), the same department (*n* = 1), or no experience in WD (*n* = 1). Ultimately, 58 responses were included in the analysis, representing 20 countries (Fig. 1).

Twenty (34%) responding centers were classified as large (≥ 30 patients per year).

The responses were collected from hepatologists (36, 62%), neurologists (5, 9%), pediatricians (7, 12%), and a mix of the above (10, 17%). Patients with hepatic and neurological affection were seen at most centers (48, 83% and 36, 62% respectively). Pediatric patients were seen at about half (30, 52%).

A large majority of responses came from tertiary medical facilities.

Below, responses to specific questions relating to diagnosis, treatment, monitoring, and screening are presented. Responses to questions not included or only partially detailed in the manuscript are available in Supplementary Table S2. Free text responses given at the end of the survey on areas of WD management in need of further research are available in Supplementary Table S3 and Supplementary Figure S4.

### Section I: diagnosing WD - adherence to guidelines and diagnostic tools

The predominant manifestation of WD among surveyed centers was hepatic. Based on the responses, the proportion of phenotypical presentations of WD was 68% hepatic, 20% neurologic, and 10% asymptomatic. Psychiatric and fulminant disease presentations were rare, both ≈ 1%.

**Fig. 2:**
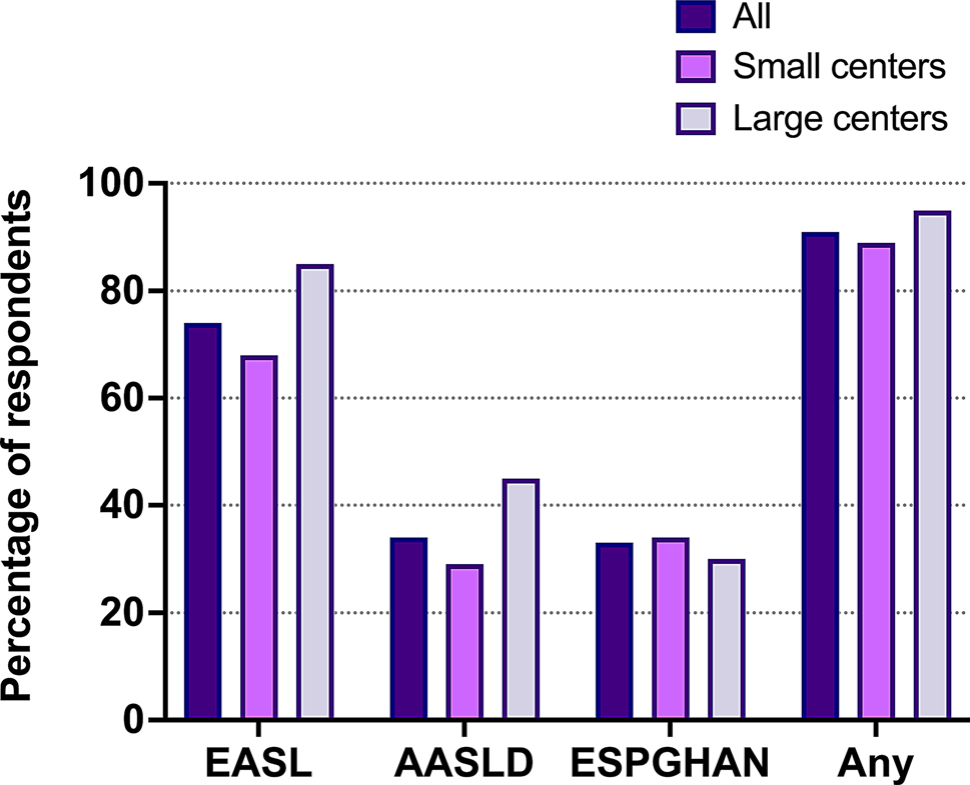
Use of international guidelines. Percentage of WD centers utilizing EASL, AASLD, or ESPGHAN international Wilson disease management guidelines in clinical practice. Data presented for all responding centers (*n* = 58), small centers (*n* = 38), and large centers (*n* = 20)

Overall, we found a high level of adherence to international guidelines by surveyed centers. Forty-three centers (74%) followed EASL guidelines, twenty (34%) AASLD and nineteen (33%) ESPGHAN guidelines, and fifty-three centers (91%) reported adherence to at least one of these. There were no clear differences between small and large centers (Fig. 2).

Fifty-one (88%) centers utilized the Leipzig criteria, again with no difference between small and large centers.

Generally, both small and large centers had sufficient tools to diagnose most cases of WD according to the Leipzig criteria, e.g., slit-lamp examinations, access to serum ceruloplasmin, urinary copper measurements, and genetic testing (Table 1). Large centers were more likely to offer more specialized, or experimental diagnostic tools, e.g., relative exchangeable copper, ^64^Cu scintigraphy, and/or calculated non-ceruloplasmin bound copper (Table 1).

**Table 1:** Diagnostic tools available at responding centers

Diagnostic tools available & used	Overall (*n* = 58)	Small centers (*n* = 38)	Large centers (*n* = 20)
24-hour urinary copper	57 (98%)	37 (97%)	20 (100%)
Serum ceruloplasmin	57 (98%)	37 (97%)	20 (100%)
Slit-lamp examination for Kayser-Fleischer rings	55 (95%)	37 (97%)	18 (90%)
Total serum copper	55 (95%)	36 (95%)	19 (95%)
Genetic testing	54 (93%)	35 (92%)	19 (95%)
Targeted mutation analysis	30 (52%)	24 (63%)	6 (30%)
Sequence analysis of entire coding region	37 (64%)	19 (50%)	18 (90%)
Haplotype analysis or only specific mutations	17 (29%)	8 (21%)	9 (45%)
Other	3 (5%)	2 (5%)	1 (5%)
Brain MRI	54 (93%)	34 (89%)	20 (100%)
Liver biopsy for histology	52 (90%)	35 (92%)	17 (85%)
Liver biopsy for copper quantification	43 (74%)	27 (71%)	16 (80%)
Penicillamine challenge test	31 (53%)	19 (50%)	12 (60%)
Calculated non-ceruloplasmin bound copper	25 (43%)	11 (29%)	14 (70%)
Relative Exchangeable Copper and Exchangeable Serum Copper	15 (26%)	7 (18%)	8 (40%)
Other tools*	4 (7%)	1 (3%)	3 (15%)
^64^copper scintigraphy	2 (3%)	0 (0%)	2 (10%)

Data presented as nn (%). *Other tools: Fibroscan

Differences were identified in the use of liver biopsies in the diagnostic process. Systematic biopsy sampling was performed in nineteen (33%) centers. Pediatric departments were more likely to use biopsies to aid diagnosis (4, 57%) compared to adult hepatological and neurological departments (12, 33% and 1, 20% respectively). Large adult hepatological departments were also more likely to use liver biopsies than small adult hepatological departments (4, 44% vs. 8, 30%).

During the diagnostic phase, most (52, 90%) centers consulted with ophthalmologists.

Neurological consultations requested by hepatological departments were prevalent (32, 89%) and systematic in larger hepatological departments (9, 100% in large vs. 23, 85% in small) and in pediatric departments (7, 100%).

The use of psychiatric and genetic consults varied substantially between centers. Just eleven (19%) centers systematically requested psychiatric consults, and twenty-one (36%) centers used genetic consults. One (2%) center did not consult other specialties.

### Section II: treating WD – treatment options and availability

**Fig. 3:**
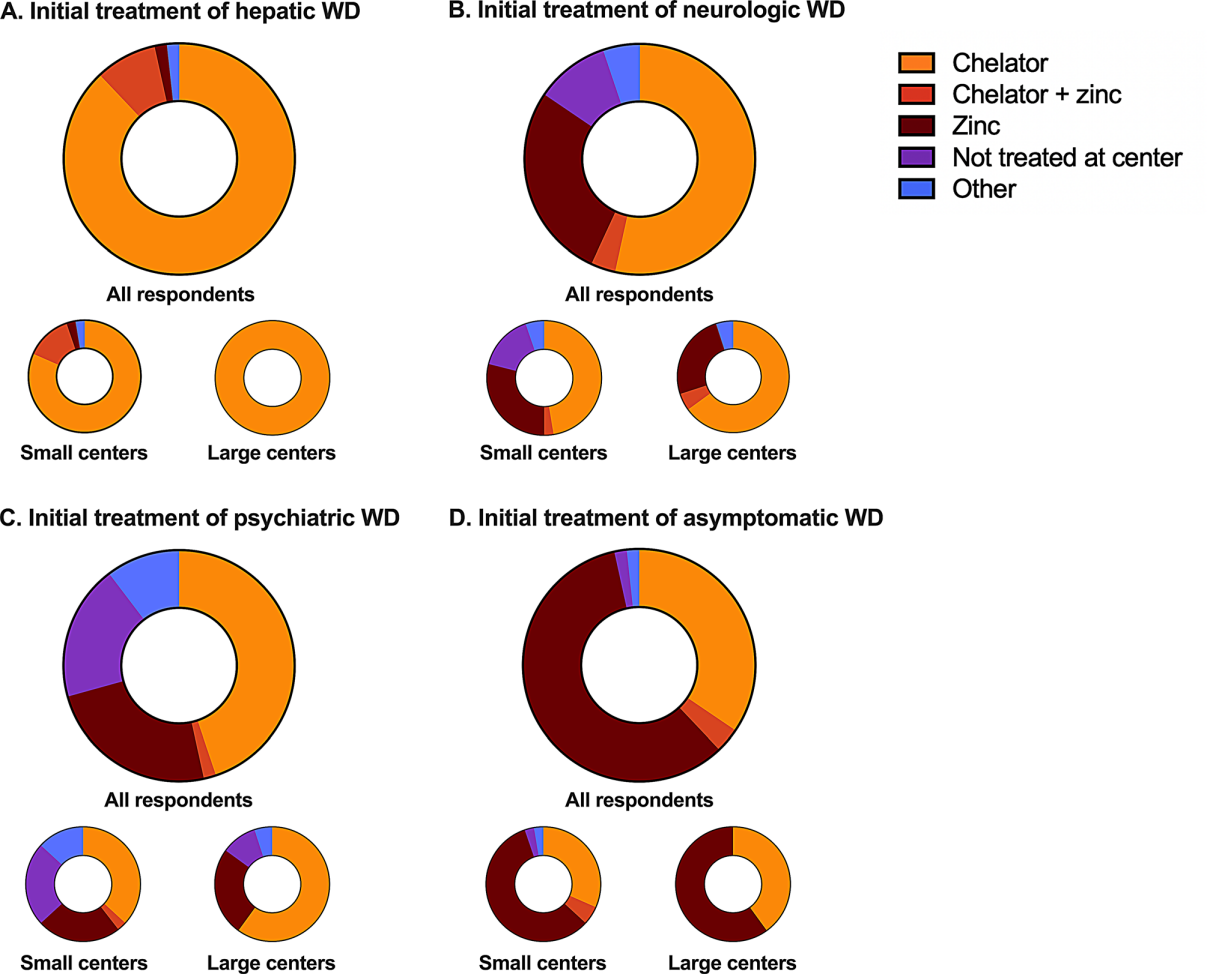
Initial treatment of Wilson disease (WD) by phenotypical presentation. (**A**) Hepatic WD. (**B**) Neurologic WD. (**C**) Psychiatric WD. (**D**) Asymptomatic WD. Data presented for all responding centers (*n* = 58), small centers (*n* = 38), and large centers (*n* = 20)

The initial therapy choice for patients with a hepatic phenotype was uniform, with chelation therapy administered alone (51, 88%) or with Zn (5, 9%) (Fig. 3).

In contrast, larger differences were identified for patients presenting with neurologic phenotype, with chelation therapy administered alone (31, 53%) or with Zn (2, 3%). Zn monotherapy was also commonly used (16, 28%). Responding neurologists were more likely to use chelation monotherapy in this setting (4, 80%).

Similar differences were found for patients with psychiatric phenotype with chelation therapy administered alone (26, 45%), with Zn (1, 2%), or Zn monotherapy (14, 24%).

Even greater differences were seen for asymptomatic patients, with chelation therapy administered alone (20, 34%), with Zn (2, 3%), or Zn monotherapy (34, 59%).

For maintenance therapy, thirty-six (62%) responding centers used chelation therapy alone or with Zn. A smaller proportion, twenty (34%), used Zn monotherapy, and two (3%) replied “other”. There were no clear differences by center size or specialization.

PEN was the most popular first-line chelator (45, 78%), over TRI (13, 22%), across both small and large centers.

**Fig. 4:**
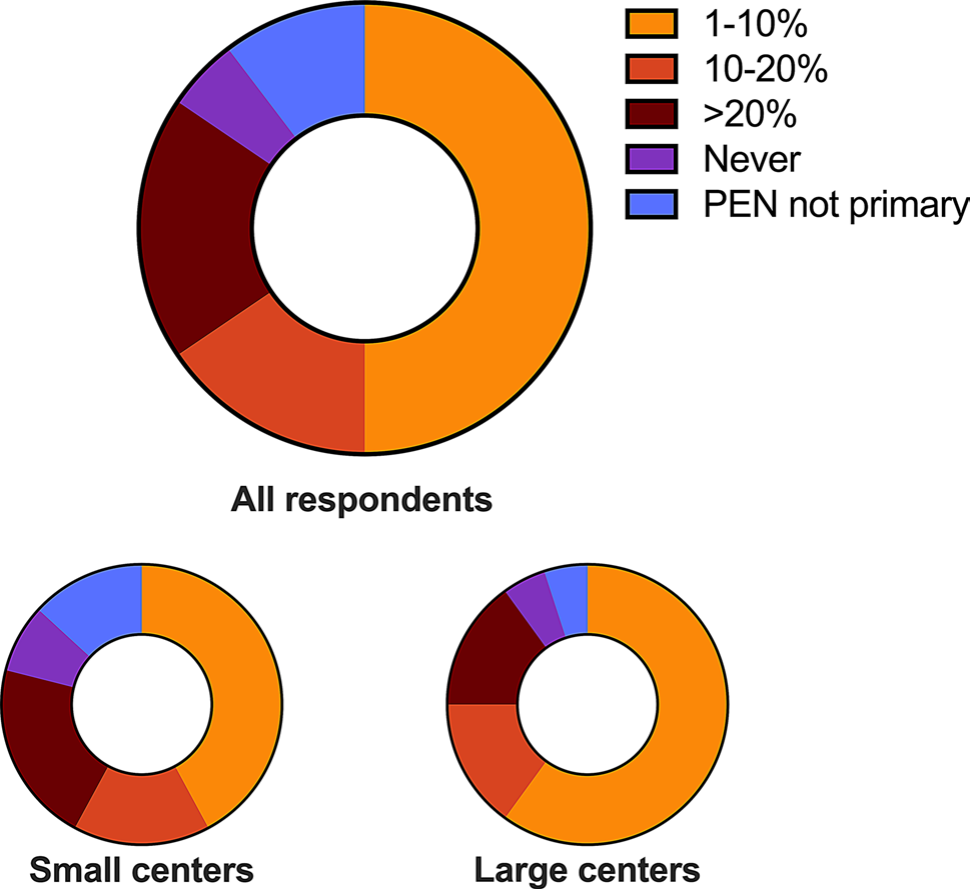
Switch from D-penicillamine due to intolerance. Proportion of respondents’ frequency of changing from D-penicillamine to other treatments over intolerance issues. Data presented for all responding centers (*n* = 58), small centers (*n* = 38), and large centers (*n* = 20)

There were clear differences across centers in how often they switched treatment due to intolerance. Thirty-two (55%) centers reported rarely or never switching from PEN to other medications, while nine (16%) did so often, and eleven (19%) very often (Fig. 4). The remaining centers did not use PEN as their initial treatment choice. The differences did not appear to relate to center size.

Different treatment options were available across most centers, but some centers lacked specific formulations. TRI was available in fifty (86%) centers. Cuprior^®^ (trientine tetrahydrochloride) was more widely available than Cufence^®^ (trientine dihydrochloride) (47, 81% vs. 28, 48%). Both TRI formulations were available in all large centers and thirty (79%) small centers.

PEN and Zn therapy were nearly universally available (56, 97% and 57, 98% respectively).

Zinc acetate was most commonly available (45, 78%). Zinc sulphate, zinc gluconate, and zinc orotate were less commonly available as reported by nine (16%), two (3%), and two (3%) responding centers, respectively.

Cost was reported to be a factor in the non-availability of different treatment modalities by four (7%) centers. All four centers were relatively small, with no access to TRI and with no clear regional similarities.

The physician-estimated rate of patient compliance varied widely across centers. Half of the respondents (29, 50%), particularly from larger centers, estimated a non-compliance rate of 25–50% while the remaining respondents estimated a lower non-compliance rate of less than 10%.

**Fig. 5:**
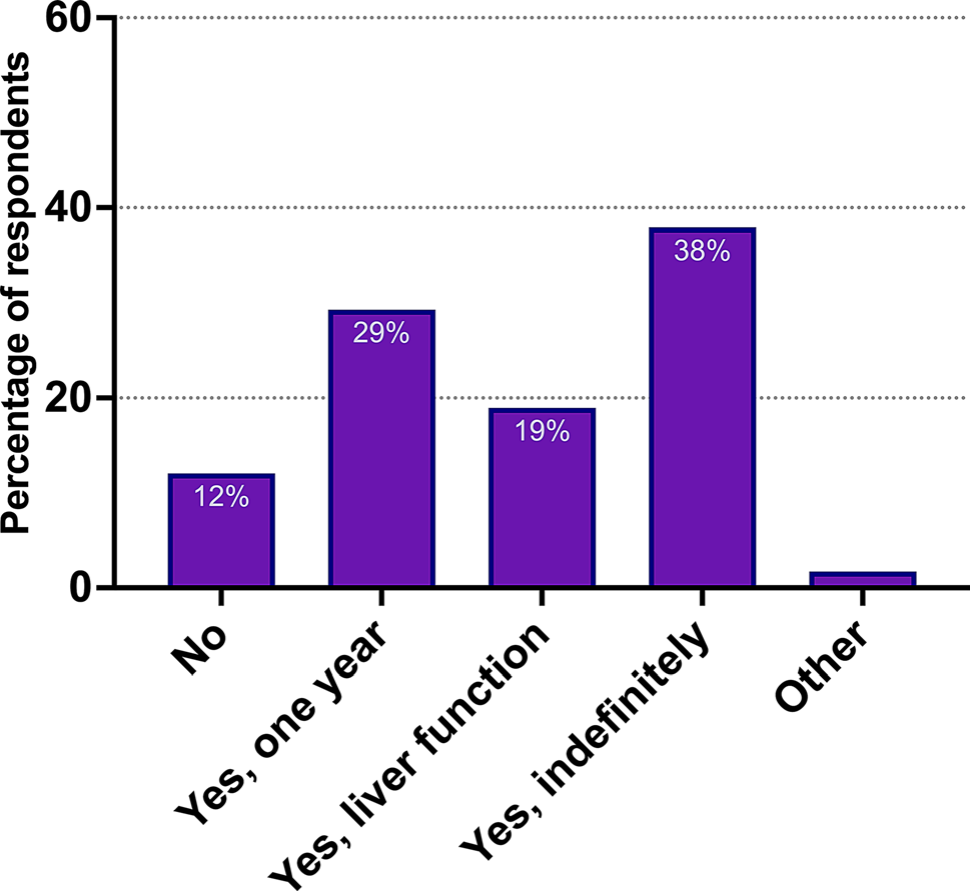
Recommendations of low copper diet for patients with Wilson disease across European centers. Those who answered yes made the decision either based on elapsed time, based on normalization of liver function tests or for indefinite low-copper diet. Data presented for all responding centers (n = 58)

Another area with major difference related to the use and form of dietary recommendations. Low copper diets were recommended by fifty-one (88%) respondents, with little difference between managing specialties (Fig. 5).

The majority (28, 48%) recommended only a temporary low-copper diet, either for one year or until normalization of liver enzymes. These results were largely unchanged by center size, but pediatric departments were more likely to suggest temporary low-copper diets (5, 71%).

### Section III: monitoring WD - treatment targets and copper-related measurements

The frequency of outpatient visits was relatively uniform across responding centers.

After reaching the treatment goal, forty-one (71%) respondents applied a 6-month frequency for visits. Most centers (49, 84%) reported that patient monitoring was more frequent before reaching the treatment goal.

A range of treatment targets were used; the question applied only to hepatic presentation of WD (Table 2). Some difference was found in the specific targets applied.

**Table 2:** Overview of responses regarding choice of treatment target in patients with WD

Treatment target	All (*n* = 58)	Hep (*n* = 38)	Ped (*n* = 7)	Neur (*n* = 5)
Normalization of liver function tests	39 (67%)	26 (72%)	6 (86%)	1 (20%)
Liver function tests less than 1.5 times UNL	22 (38%)	13 (36%)	1 (14%)	4 (80%)
24 H-UCE of approximately 200–500 µg or 3–8 µmol on maintenance chelation therapy	38 (66%)	25 (69%)	4 (57%)	2 (40%)
Free serum copper 5–15 µg/dL or 50–150 µg/L	19 (33%)	12 (33%)	1 (14%)	3 (60%)
Regression of Kayser-Fleischer rings	15 (26%)	9 (25%)	1 (14%)	2 (40%)

Presented for all respondents and by specialization. Hep = Hepatological department. Ped = Pediatric department. Neur = Neurological department. 24 H-UCE = 24-hour urinary copper excretion. UNL = Upper Normal Limit

Normalization of liver function tests and rise of 24 H-UCE from baseline on maintenance chelation therapy were used by thirty-nine (67%) and thirty-eight (66%) respondents. Normalization of liver function tests was more commonly reported by hepatologic (26, 72%) and pediatric departments (6, 86%) compared to neurological departments (1, 20%).

Instead, liver function tests being less than 1.5 times the upper normal limit was the preferred hepatic treatment target in neurological departments.

The most widely preferred copper-related measurement used for monitoring treatment was 24 H-UCE, as reported by fifty-seven (98%) respondents. This was followed by cNCC (15, 26%) and CuEXC (12, 21%). No clear differences were seen between small and large centers or by specialization.

Some key differences were identified in the pre-analytical sampling of urine analysis. When collecting 24 H-UCE, forty-one (71%) centers did not pause chelation therapy prior to collection. Three centers (5%) paused chelation therapy for one day before urine collection, and seven (12%) centers paused chelation therapy for three days before urine collection.

Of particular note, there were also differences found regarding the use of percutaneous liver biopsies in the maintenance phase of WD management, when non-invasive markers were insufficient to evaluate disease control. Twelve (21%) centers reported performing liver biopsies in such cases during the maintenance phase, particularly large as opposed to small centers, six (30%) vs. six (16%). No clear differences were found between different specialties.

### Section IV: patient organization and family screening

Collaboration between WD centers and Patient organizations (PTO) varied widely. Twenty-two centers (38%) indicated minimal contact with PTOs, while eighteen (31%) reported the absence of a PTO in their country. A minority (11, 19%) reported consistent communication and collaboration with a highly active PTO, and seven (12%) had an active PTO, but collaboration was not regular.

Family screening was offered almost universally, with genetic testing more commonly used compared to biochemical analysis of blood and urine alone (48, 83% vs. 9, 16%).

## Discussion

In this cross-sectional study, a physician-oriented internet survey was developed and delivered through collaboration within the ERN-RARE Liver Wilson working group with the aim of studying the management of WD across the treating centers. This is the first major pan-European survey on Wilson Disease management involving adult and pediatric centers.

**Fig. 6:**
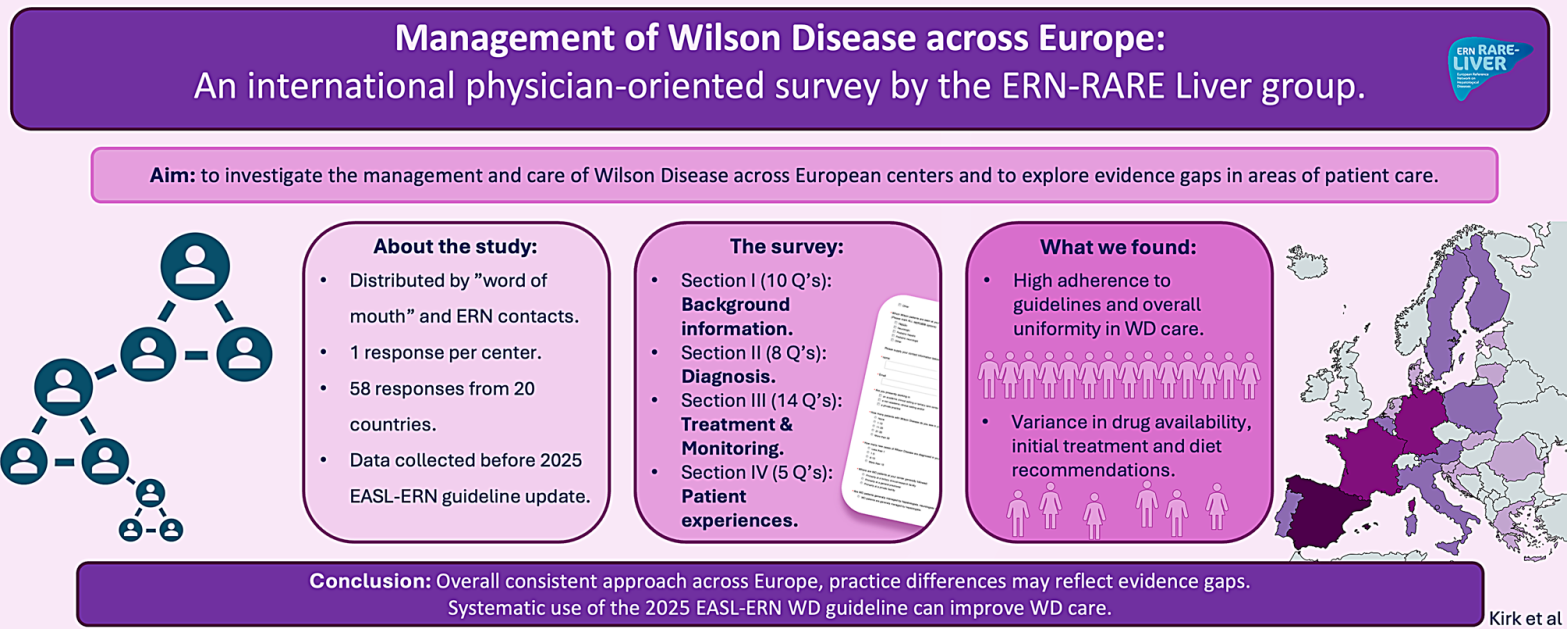
Visual presentation of the study. The overall aims, methods, results and conclusions based on this study are presented visually

Overall, the study showed a relatively high degree of uniformity in the management of WD across the European centers that responded, and in particular, adherence to international guidelines, the use of the Leipzig criteria for the diagnosis of WD, as well as the initial treatment of patients with WD with hepatic phenotype (Fig. 6).

The survey also revealed notable variations in WD management across European centers, highlighting areas lacking strong evidence and potential for improved standardization. These differences were often somewhat more pronounced in smaller centers, defined as managing < 30 patients - a threshold based primarily on adult patient patterns in countries with or without centralization policies. Therefore, the responses may indicate a potential effect of centralization.

Important differences in the diagnostic phase of WD management were noted.

One such difference involved the use of liver biopsy sampling in the diagnostic phase of WD management. Liver biopsy entails risks, but hepatic copper quantification and fibrosis evaluation provide diagnostic information and may guide management. Liver biopsies may be more critical in pediatric patients, where neurological symptoms, MRI changes, and Kayser-Fleischer rings are less common. In contrast, patients with neurological symptoms may be less likely to require a liver biopsy to achieve a diagnostic Leipzig score. The information obtained from liver biopsy samples is still not accessible by non-invasive markers. Until further research has provided this evidence, liver biopsy remains an important but limited tool [17–19].

Most centers did have sufficient diagnostic tools available, however, direct non-ceruloplasmin bound copper measurements (e.g., CuEXC) was unavailable to most (Table 2). CuEXC has shown promise as a tool to monitor treatment effects, and when normalized to total serum copper has high diagnostic accuracy [13, 20–23]. The low availability of CuEXC likely reflects that it is a relatively new tool and was only adopted by the EASL guideline in 2025 [2].

The survey highlighted the low utilization of psychiatric and genetic consults in the diagnostic phase. The 2012 EASL guidelines do not make recommendations for consulting other specialties [16]. However, the 2022 AASLD guideline recommends consulting psychiatrists for screened patients [1] and the 2025 EASL guideline recommends evaluation of neuropsychiatric affection in patients with WD [2]. The data on consults suggested that joint management of patients between hepatologists and neurologists was not systematic in smaller centers.

Thus, the depicted consulting patterns reveal a possible opportunity for better patient management.

Notable differences in the treatment of WD were also identified.

These differences included the initial treatment of patients with WD with non-hepatic phenotype, particularly those with neurologic phenotype. Chelation therapy was used by approximately half of the respondents for neurologic and psychiatric WD, and approximately one-third of asymptomatic patients. The difference for neurologic WD was less pronounced amongst neurological departments. This was surprising given that guidelines at the time of data collection recommended chelation therapy for initial management of WD [1]. Zn has been favored by some, but controversy exists regarding the use of initial Zn therapy [24–28]. The controversy in responses may relate to differences in definitions of asymptomatic patients (Whether this includes alanine aminotransferase elevations or not). Nonetheless, the 2025 EASL guideline update recommends Zn or chelation therapy for patients with neurologic phenotype and asymptomatic patients (without significant liver injury). Whilst guidelines have changed to better support the practice reported by the respondents, our findings highlight that stronger evidence is needed, and the importance of understanding guideline adherence patterns in clinical practice.

Notably, combination therapy was used by 9% for the standard initial treatment of Wilson Disease with hepatic phenotype. Combination therapy is not unheard of, but it is considered off-label use, is not generally recommended by international guidelines, and is not clearly supported by the limited available evidence [1, 2, 29].

Another point of difference was found in the availability of different pharmacological treatments. Specifically, 14% of centers, all small, had no access to TRI (trientine dihydrochloride and tetrahydrochloride). Cost was a factor according to half of the respondents. Interestingly, the newer TRI formulation Cuprior^®^ was more widely available than Cufence^®^.

Low copper diet recommendations were also debatable amongst respondents. That is, approximately half of the respondents recommended low-copper diet for a limited time, a large minority recommended low-copper diet indefinitely, and a smaller group had no recommendations. Only the AASLD guideline had dietary recommendations at the time of data collection, but the updated 2025 EASL guideline now recommends, through consensus, a temporary copper-restricted diet. The responses likely reflect the lack of strong evidence, with reviews suggesting low-copper diet to have little to no effect, while negatively impacting quality of life [30–32].

There was a surprisingly high variability in physician-estimated rate of patient compliance. Given the importance of adherence for WD patients and reports of low adherence in literature, a systematic and standardized approach to its evaluation, e.g., recurrent questionnaires, could be implemented in future guideline updates [33, 34].

This study also uncovered differences in the monitoring of patients with WD.

Most centers continued treatment during the collection of 24 H-UCE samples for monitoring treatment. A minority paused treatment for 1–3 days prior to collection. Evidence as to which method should be preferred is lacking, but 24 H-UCE after a treatment-pause might better reflect the bioavailable copper pool, and 24 H-UCE on-treatment may better reflect adherence to treatment [35, 36]. Nonetheless, 24 H-UCE sampling has many limitations, and care is required in the interpretation. Furthermore, it must be reinforced that laboratory monitoring parameters (e.g., CuEXC and 24 H-UCE) have not been appropriately validated vs. clinical outcomes thus far, though there is emerging supporting data [20, 21].

Differences in the treatment target for liver enzymes were also noted, with hepatologists preferring normalization of these enzymes, while neurologists were more likely to target levels 1.5 times the upper normal limit. The 2012 EASL guideline did not establish target levels, whilst the recent 2025 EASL-ERN guideline indicates that normalization of liver enzymes indicates a complete liver response [2, 16]. While liver enzymes should not be used alone to predict disease progression, lack of normalization may be associated with worse outcomes [37].

Lastly, the study highlights differences in collaborations between centers and PTO’s, revealing an opportunity for improvement. PTOs are important resources for patients with WD, including families and carers, especially those newly diagnosed. PTO’s can play an important role in developing networks between patients and the public, reducing anxiety and non-adherence, as well as increasing involvement and engagement in research participation. Active PTO collaboration is important in both clinical and research settings [38–41].

Stronger PTO collaborations may also impact areas such as dietary recommendations, which are lacking in strong evidence, but may severely impact quality of life.

Few physician-oriented surveys on WD management exist. Sturm et al. reported on pediatric care, with largely similar findings to ours, except for less frequent switching from PEN, likely due to age differences [42]. Zimny et al. surveyed German centers and also found comparable practices, though with lower use of the Leipzig criteria, more frequent follow-ups, and more common pausing of chelation before 24 H-UCE collection— reflecting a decentralized care structure in Germany [43].

This study has limitations. The survey was distributed via the ERN RARE-Liver network, which may not have reached all European WD centers, potentially introducing selection bias—highlighted by the broader participation seen in a recent German survey [43]. WD’s heterogeneity may have led to misinterpretation of some questions, particularly regarding initial treatment, as no case vignettes were provided. The relatively low representation of neurologists may also have biased results, this issue could be due to the survey’s liver-focused origin or simply due to the predominance of WD care in hepatological departments. Moreover, pediatric centers typically see fewer patients than corresponding adult centers, simply because of age distribution. This could in turn impact our classification of center size. The overrepresentation of Spanish centers may have biased the overall results, but so would the censoring of eligible responses. The reason for this overrepresentation may be a more decentralized approach, the 2021 national AEEH WD registry initiation, or in part, simply the large geography and population. Physicians responded based on recollection rather than chart review, which may have affected the results; however, as questions were relatively general, this likely had little effect.

Finally, updated AASLD and EASL guidelines were published after survey distribution [1, 2] and thus could not be included in the survey, but our findings still reflect current variations in practice across Europe.

As international clinical trials in WD increase, our findings underscore significant variability in diagnosis, treatment, and monitoring across European centers. These differences should be accounted for in study design and clinical interpretation.

## Conclusions

In conclusion, this physician-oriented survey shows a relatively high degree of uniformity in the management of WD across European centers. The survey uncovers important differences among centers, particularly related to the initial treatment of non-hepatic WD, availability of TRI, and recommendations for low-copper diet. The survey highlights numerous areas in WD care in which robust evidence is lacking, and which should be considered in designing future multi-collaborative international clinical trials.

## Supplementary Information

Below is the link to the electronic supplementary material.

Supplementary Material 1

Supplementary Material 2

## Data Availability

The datasets used and/or analysed during the current study are available from the corresponding author on reasonable request.

## Abbreviations

WD: Wilson Disease
TRI: Trientine
PEN: D-penicillamine
ZN: Zinc
cNCC: Calculated Non-ceruloplasmin bound copper
CuEXC: Exchangeable serum copper
24H-UCE: 24-hour urinary copper excretion
ERN-RARE Liver: European Reference Network on Rare Liver Disorders
PTO: Patient organization
