# Supplementary material for: Management of Wilson disease across Europe: an international physician-oriented survey by the ERN-RARE Liver group
Source: Orphanet J Rare Dis. 2025 Nov 11;20:573. doi: 10.1186/s13023-025-04103-6 (PMC12607149; doi:10.1186/s13023-025-04103-6)
Supplement: Supplementary file 1 — Supplementary Material 1 [file 13023_2025_4103_MOESM1_ESM.pdf]

survey questions for evaluation of clinical practice in Wilson Disease across ERN centers.

Fields marked with * are mandatory.

Goal:
We wish to map the current clinical practice in treatment, diagnosis, and management as well as patient perspectives in Wilson Disease (WD) across Europe,
Ultimately, we wish to describe equality of WD care across Europe (in line with the ERN perspective).

If data are published, the paper will have the authorship of the core group, who will write the paper, and as final author the name of the (group) lead of the ERN RARE-LIVER, and all individual contributors will then be listed as part of this group or included in the acknowledgements.

Background:

How many ERN centres manage WD patients?
Are WD patients primarily seen in primary, secondary or tertiary centres or at GP level?
How specialized in WD are the ERN centres?

Diagnosis:

Which diagnostic tools exists across ERN centres?
Which diagnostic tools are preferred for initial evaluation?
How is genetic analysis and liver biopsy pathology used for the diagnosis of WD?
Which specialists see WD patients during the diagnostic process?

Treatment:

Which medications are first choice in (Stable, acute, fulminant) WD across ERN centres?
Which treatment options are available - and what role does cost play in availability?
Is combination therapy used in WD treatment, and what are the indications?
How is WD treatment transitioned from pediatric to adult cases?
Is multidisciplinary treatment of WD, if needed, guaranteed?

Patient perspectives:

How closely are WD patients monitored?

What is the general compliance-rate of WD patients across ERN centres?
Do centres collaborate with patient`s associations/ do they connect patients with disease specific associations?

1. Background information on replying physician and medical center:

* In which country do you practice?

- [ ] Austria
- [ ] Belgium
- [ ] Bulgaria
- [ ] Croatia
- [ ] Republic of Cyprus
- [ ] Czech Republic
- [ ] Denmark
- [ ] Estonia
- [ ] Finland
- [ ] France
- [ ] Germany
- [ ] Greece
- [ ] Hungary
- [ ] Ireland
- [ ] Italy
- [ ] Latvia
- [ ] Lithuania
- [ ] Luxembourg
- [ ] Malta
- [ ] Netherlands
- [ ] Poland
- [ ] Portugal
- [ ] Romania
- [ ] Slovakia
- [ ] Slovenia
- [ ] Spain
- [ ] Sweden
- [ ] Norway
- [ ] Switzerland
- [ ] Other

* Please name the center you are answering on behalf of

* What is the specialization of the department you are answering on behalf of

- [ ] Hepatology (& Gastroenterology)
- [ ] Neurology
- [ ] Pediatric

- [ ] Other

* Which Wilson patients are seen at your center?
(Please mark ALL applicable options)

- [ ] Hepatic
- [ ] Neurologic
- [ ] Pediatric hepatic
- [ ] Pediatric neurologic
- [ ] Other

Please supply your contact information below

* name:

____

* Email

____

* Are you presently working in

- [ ] an academic clinical setting or tertiary care center
- [ ] a non-academic clinical setting and/or
- [ ] a private practice

* How many patients with Wilson Disease do you see in your practice during one year?

- ( ) None
- ( ) 1-10
- ( ) 11-20
- ( ) 21-30
- ( ) More than 30

* How many new cases of Wilson Disease are diagnosed in your center per year?

- ( ) Less than 1
- ( ) 1-5
- ( ) 6-10
- ( ) More than 10

* Where are WD patients at your center generally followed:

- ( ) Primarily at a tertiary clinical/research facility
- ( ) Primarily at a general practioner
- ( ) Primarily at a private facility

* Are WD patients generally managed by hepatologists, neurologists or jointly?

- ( ) WD patients are generally managed by hepatologists.

- WD patients are generally managed by neurologists.
- WD patients are generally managed jointly by hepatologists and neurologists.

2. Approach to diagnosis

* Which diagnostic tools are available at your center?
Please mark ALL relevant options:

- Liver biopsy **with** quantification of copper content in liver tissue
- Liver biopsy **with** histopathological analysis of liver tissue
- Baseline copper in 24-h urine
- Slit-lamp examination for Kaiser Fleischer rings
- Serum ceruloplasmin
- Total serum copper
- Non-ceruloplasmin bound copper (NCC)
- Relative Exchangeable Copper (REC) & Exchangeable Serum Copper (CuEXC)
- Penicillamine challenge test
- Genetic testing
- Scintigraphy 64Cu for Cu-incorporation in ceruloplasmin
- Brain MRI
- Others

* Is Leipzig (Ferenci) scoring used for Wilson Disease diagnosis at your center?

- Yes
- No

* If you do not use Leipzig (Ferenci) scoring, which method is used for Wilson Disease diagnosis at your center?

- AASLD practice guideline update (Roberts and Schilsky, Hepatology 2008; 47: 2089-2111)
- Criteria as described in Rosencrantz and Schilsky, Seminars in Liver Disease 2011; 31: 245-259
- Regional criteria/guideline. Please specify below:
- Clinical assessment
- Other

* When diagnosing Wilson Disease, which other specialists see the patients?
Please mark all that apply

- Ophthalmologists
- Neurologists
- Psychiatrists
- Geneticists
- None
- Other

* In which cases do you perform liver biopsy with a determination of liver copper content? Please mark ONE, TWO, OR THREE items:

- Only in selected cases, when the diagnosis is uncertain
- Systematically at diagnosis to have a starting point.

- [ ] During follow-up, when non-invasive markers are judged to be insufficient to evaluate the disease control.
- [ ] Not available where I practice
- [ ] Other cases

* How do you use genetic testing in diagnosing WD?
Please choose ONE OR MORE options that seem appropriate:

- [ ] Although available, I do not use it, standard clinical tests are sufficiently reliable
- [ ] It is not available where I practice
- [ ] I use it in selected cases
- [ ] I use it in all suspected cases
- [ ] I use it also to confirm diagnosis
- [ ] Namely:

* Which type of genetic testing is performed in your (or collaborating) lab?
Please choose ONE OR MORE options that seem appropriate:

- [ ] We screen for the most prevalent mutations in the patient's population: targeted mutation analysis
- [ ] Sequence analysis of the entire coding region
- [ ] We perform haplotype analysis or DNA analysis for specific mutations when screening an affected family
- [ ] Other

How many percent % of your patients with Wilson Disease presented with the following primary symptoms

(please make sure that all percentages add up to 100% total and all fields must be filled with a number between 0 and 100%)

primary symptom	percent
* Hepatic	
* Neurologic	
* Psychiatric	
* Asymptomatic	
* Fulminant	

3. Approach to treatment

* Which WD MANAGEMENT guideline(s) do you use in your practice?

- [ ] None
- [ ] AASLD practice guideline update (Roberts and Schilsky, Hepatology 2008; 47: 2089-2111)
- [ ] As advised in Rosencrantz and Schilsky, Seminars in Liver Disease 2011; 31: 245-259
- [ ] EASL practice guideline (Journal of Hepatology 2012)
- [ ] ESPGHAN 2018 Position paper on Wilson Disease in children (Socha, Hepatology and Nutrion 2018)
- [ ] A regional guideline
- [ ] Other

* Do you advice newly diagnosed Wilson Disease patients to adhere to a low copper diet (<1 mg per day)?

- ○ No
- ○ Yes, in the first year of treatment

- Yes, until normalization of liver function tests
- Yes, indefinitely
- Other

* What is the standard INITIAL treatment for HEPATIC Wilson Disease patients with significant liver disease in your center?

- Chelator
- Chelator + Zinc
- Zinc
- Our center does not treat hepatic Wilson patients
- Other

* What is the standard INITIAL treatment for NEUROLOGICAL Wilson Disease patients in your center?

- Chelator
- Chelator + Zinc
- Zinc
- Our center does not treat neurologica Wilson patients
- Other

* What is the standard INITIAL treatment for PSYCHIATRIC Wilson Disease patients in your center?

- Chelator
- Chelator + Zinc
- Zinc
- Our center does not treat asymptomatic Wilson patients
- Other

* What is the standard INITIAL treatment for ASYMPTOMATIC Wilson Disease patients in your center?

- Chelator
- Chelator + Zinc
- Zinc
- Our center does not treat asymptomatic Wilson patients
- Other

* What is the standard MAINTENANCE treatment for Wilson Disease patients at your center?

- Chelator
- Chelator + Zinc
- Zinc
- Other

* What is your first-line choice of chelator?

- Trientine
- Penicillamine
- Other

* Which formulation of zinc do you usually prescribe?

- [ ] Zinc acetate
- [ ] Zinc sulphate
- [ ] Zinc gluconate
- [ ] Zinc orotate
- [ ] I do not prescribe zinc for treating Wilson Disease

* Which treatment options are available at your facility?

- [ ] Trientine dihydrochloride (e. g. CUFENCE)
- [ ] Trientine tetrahydrochloride (e. g. CUPRIOR)
- [ ] Penicillamine
- [ ] Zinc
- [ ] Other
- [ ] PLEASE MARK THIS IF COST IS A REASON WHY ONE OPTION IS UNAVAILABLE

* What is your target of therapy when treating HEPATIC WD?
Please mark ALL appropriate answers:

- [ ] Normalization of liver function tests in the first year of treatment
- [ ] Liver function tests are less than 1,5 times the upper limit of normal in the first year of treatment
- [ ] Copper in 24-h urine rises from baseline to ca. 200-500 µg or 3-8 µmol on maintenance treatment with trientine or D-penicillamine for the first months of therapy
- [ ] Free serum copper 5-15 µg/dl or 50-150 µg/l
- [ ] Kayser-Fleischer ring regression
- [ ] Other

* When measuring 24-h urinary copper, is chelation therapy paused prior to urinary collection?

- ○ Yes, chelation therapy is paused 1 day prior to urinary collection
- ○ Yes, chelation therapy is paused 3 days prior to urinary collection
- ○ No, chelation therapy is continued during urinary collection
- ○ Other

* How often did you have to change from D-penicillamine as the primary medication to an alternative due to undesired side effects?

- ○ I don´t use D-penicillamine as primary therapy
- ○ Never
- ○ In 1 out of 100 to 1 out of every 20 patients
- ○ In 1 of 19 to 1 out of every 10 patients
- ○ In 1 of 9 to 1 out of every 5 patients
- ○ In more than 1 in 5 patients

* What is the preferred copper related measurement to monitor treatment in Wilson patients at your center?
Please mark ONE, TWO, THREE or FOUR options

- [ ] 24-h urine copper
- [ ] Non-Ceruloplasmin bound Copper (NCC)
- [ ] Exchangeable Serum Copper (CuEXC)
- [ ] Other

* When a stable Wilson patient is transferred from a pediatric center / department to you, is medication generally changed?

- Patient care is not transferred from pediatric centers / departments to our center.
- In general, patient treatment is not changed.
- In general, all patients are put on chelation therapy using TRIENTINE
- In general, all patients are put on chelation therapy using PENICILLAMINE
- In general, all patients are put on zinc therapy

4. Patient perspectives

* How often are Wilson patients seen at your center before the treatment goal has been reached?

- More often than once a month
- More often than once every 6 months
- Every 6 months
- Every 12 months
- Less often than every 12 months

* How often are STABLE Wilson patients seen at your center OR?

- More often than every 6 months
- Every 6 months
- Every 12 months
- Less often than every 12 months
- Stable Wilson patients are not seen at our center, but are followed elsewhere
- Stable Wilson patients are not seen regularly

* By your estimate, what is the rate of non-compliance in treatment for Wilson patients at your center?

- Less than 10% are non-compliant in treatment
- 25% are non-compliant in treatment 50% are non-compliant in treatment
- 75% are non-compliant in treatment
- More than 75% are non-compliant in treatment

* When diagnosing Wilson Disease, are family members offered screening? (please mark ALL applicable answers)

- Yes, genetic screening is offered to immediate family members
- Yes, standard biochemistry including copper measurements (blood+urine).
- No, we do not offer screening of asymptomatic family members

* Do you collaborate with patient´s organisations?

- no patient association in our country
- Very little substantial contact with PO
- Active PO with some but not regular collaboration
- Very active PO with regular contact and joint meetings.

5. Your comments

Please enter general comments and feedback on this survey here:

Which questions in diagnosing and treating WD need further investigation?

Contact

Contact Form
