# Supplementary material for: Management of Wilson disease across Europe: an international physician-oriented survey by the ERN-RARE Liver group
Source: Orphanet J Rare Dis. 2025 Nov 11;20:573. doi: 10.1186/s13023-025-04103-6 (PMC12607149; doi:10.1186/s13023-025-04103-6)
Supplement: Supplementary file 2 — Supplementary Material 2 [file 13023_2025_4103_MOESM2_ESM.docx]

**Supplementary materials**

**S1: Supplementary file “The survey as distributed to respondents”.**

Please see separate file.

**S2: Supplementary table S2: Additional survey data.** Data is given as n (%) from the overall respondents and, for brevity, not reported by subgroups unless otherwise specified. Data included is either not reported or partially reported in the manuscript.

| **Background information on replying physician and medical center:** | |
| --- | --- |
| **In which country do you practice?** | Austria : 3 (5%)  2 classified as large  Belgium : 2 (3%)  Croatia : 1 (2%)  Denmark : 1 (2%)  1 classified as large  Estonia : 1 (2%)  Finland : 2 (3%)  France : 6 (10%)  2 classified as large  Germany : 8 (14%)  4 classified as large  Greece : 1 (2%)  1 classified as large  Italy : 3 (5%)  2 classified as large  Lithuania : 1 (2%)  Luxembourg : 1 (2%)  Netherlands : 1 (2%)  1 classified as large  Poland : 2 (3%)  2 classified as large  Portugal : 2 (3%)  1 classified as large  Romania : 1 (2%)  Slovakia : 1 (2%)  Slovenia : 1 (2%)  Spain : 18 (31%)  3 classified as large  Sweden : 2 (3%)  1 classified as large |
| **Are you presently working in** | An academic clinical setting or tertiary care setting : 54 (93%)  A non-academic clinical setting : 2 (3%)  A Private practice : 2 (3%) |
| **How many patients with Wilson Disease do you see in your practice during one year?** | None : 0 (0%)^*^  1-10 : 21 (36%)  11-20 : 10 (17%)  21-30 : 7 (12%)  More than 30 : Reported in results  ^*^ Responses from physicians not involved with WD care were excluded from the analysis (n=1). |
| **How many new cases of Wilson Disease are diagnosed in your centre per year?** | Less than 1 : 18 (31%)  1-5 : 36 (62%)  6-10 : 2 (3%)  More than 10 : 2 (4%) |
| **Where are WD patients at your centre generally followed:** | Primarily at a tertiary clinic/research facility : 56 (97%)  Primarily at a general practioner : 0 (0%)  Primarily at a private facility: 2 (3%) |
| **Are WD patients generally managed by hepatologists, neurologists or jointly?** | WD patients are generally managed by hepatologists : 29 (50%)  WD patients are generally managed by neurologists : 0 (0%)  WD patients are generally managed jointly by hepatologists and neurologists : 29 (50%) |
| **Approach to diagnosis:** | |
| **If you do not use Leipzig (Ferenci) scoring, which method is used for Wilson Disease diagnosis at your centre?** | Seven (12%) reported not using the Leipzig criteria. Responses from these seven physicians are reported below (more than one option could be chosen):  AASLD 2008 guideline^*^ : 5  Rosencrantz and Schilsky, Seminars in Liver Disease 2011 : 1  Regional guideline : 1  Clinical assessment : 1  ^*^ The survey was created prior to the 2022 AASLD guideline update. |
| **When diagnosing Wilson Disease, which other specialists see the patients? Please mark all that apply** | Data presented in the results section.  Additionally, seven (12%) of participants reported consulting “other” specialties. |
| **In which cases do you perform liver biopsy with a determination of liver copper content? Please mark ONE, TWO, OR THREE items:** | Data presented is n (%) of all responses. Further subgrouping was omitted for brevity.  Only in selected cases, when the diagnosis is uncertain   - 37 (64%)   Systematically at diagnosis to have a starting point   - See results section   During follow-up, when non-invasive markers are judged to be insufficient to evaluate the disease control   - 12 (21%)   Not available where I practice   - 0 (0%)   Other cases   - 0 (0%) |
| **How do you use genetic testing in diagnosing WD? Please choose ONE OR MORE options that seem appropriate:** | *This is partly answered in the main manuscript.*  Although available, I do not use it, standard clinical tests are sufficiently reliable : 2 (3%)  It is not available where I practice : 1 (2%)  I use it in selected cases : 7 (12%)  I use it in all suspected cases : 42 (72%)  I use it also to confirm diagnosis : 32 (55%)  Respondents selecting the “I use it in selected cases”:  5: In uncertain cases  1: When requested by patients |
| **Approach to treatment:** | |
| **Which WD MANAGEMENT guideline(s) do you use in your practice?** | In addition to those results presented in section I of the results:  Option selected : n : % of all  None : 2 : 3%  Rosencrantz and Schilsky, Seminars in Liver Disease 2011 : 3 : 5%  Regional guideline : 3 : 5%  Other : 4 : 7% |
| **What is the standard INITIAL treatment for HEPATIC Wilson Disease patients with significant liver disease in your centre?** | *A selection of the data below is also given in the results section.*  Chelator : 51 (88%)  Chelator + Zinc : 5 (9%)  Other : 1 (2%)  Zinc : 1 (2%) |
| **What is the standard INITIAL treatment for NEUROLOGICAL Wilson Disease patients in your centre?** | *A selection of the data below is also given in the results section.*  Chelator : 31 (53%)  Chelator + Zinc : 2 (3%)  Other : 3 (5%)  Zinc : 16 (28%)  Our centre does not treat neurological patients : 6 (10%) |
| **What is the standard INITIAL treatment for PSYCHIATRIC Wilson Disease patients in your centre?** | *A selection of the data below is also given in the results section.*  Chelator : 26 (45%)  Chelator + Zinc : 1 (2%)  Other : 6 (10%)  Zinc : 14 (24%)  Our centre does not treat psychiatric patients : 11 (19%) |
| **What is the standard INITIAL treatment for ASYMPTOMATIC Wilson Disease patients in your centre?** | *A selection of the data below is also given in the results section.*  Chelator : 20 (34%)  Chelator + Zinc : 2 (3%)  Other : 1 (2%)  Zinc : 34 (59%)  Our centre does not treat asymptomatic patients : 1 (2%) |
| **What is the standard MAINTENANCE treatment for Wilson Disease patients at your centre?** | *Data is given in the results section.*  *Of 36 centres using chelation +/- Zinc, 3 centres did use zinc and 33 chelation alone.* |
| **What is your target of therapy when treating HEPATIC WD? Please mark ALL appropriate answers:** | This is partly answered in the main manuscript.  Normalization of liver function tests in the first year of treatment Liver function tests are less than 1,5 times the upper limit of normal in the first year of treatment  Copper in 24-h urine rises from baseline to ca. 200-500 μg or 3-8 μmol on maintenance treatment with trientine or D-penicillamine for the first months of therapy Free serum copper 5-15 μg/dl or 50-150 μg/l Kayser-Fleischer ring regression  Other |
| **When a stable Wilson patient is transferred from a pediatric centre / department to you, is medication generally changed?** | Patient care is not transferred from pediatric centres / departments to our centre : 4 (7%)  In general, patient treatment is not changed : 51 (88%) In general, all patients are put on chelation therapy using TRIENTINE : 0 (0%) In general, all patients are put on chelation therapy using PENICILLAMINE : 2 (3%)  In general, all patients are put on zinc therapy : 1 (2%) |
| **Patient perspectives:** | |
| **How often are Wilson patients seen at your centre before the treatment goal has been reached?** | *This is partly answered in the main manuscript.*  More often than once a month : 2 (3%)  More often than every 6 months : 47 (81%)  Every 6 months : 8 (14%)  Every 12 months : 1 (2%)  Less often than every 12 months 0 (0%) |
| **How often are STABLE Wilson patients seen at your centre OR?** | *This is partly answered in the main manuscript.*  More often than every 6 months : 3 (5%)  Every 6 months : (41 (71%)  Every 12 months : 13 (22%)  Less often than every 12 months : 1 (2%)  Stable Wilson patients are not seen at our centre, but are followed elsewhere : 0 (0%)  Stable Wilson patients are not seen regularly : 0 (0%) |
| **Your comments:** | |

**S3: Supplementary table “**Answers to the question, ‘**Which questions in diagnosing and treating WD need further investigation?’ ”**:

|  | **Comments:** |
| --- | --- |
|  | Better biomarkers, better drugs with effect on neurology |
|  | Till now is no single marker for diagnosis and monitoring. |
|  | Pregnancy and WD |
|  | management of refractory neurological symptoms |
|  | What is the best strategy to assess adequate treatment response?  How can Fulminant hepatic failure be prevented?  How can recovery from acute liver failue be predicted?  What is the best management stratgy for neuro Wilson  LTx for neurowilson?  non ATP7B Wilson - genetic cause?? |
|  | ExCu or NCC should be included in the regular follow-up of patients (in Spain it is not done) |
|  | Best tools for patient monitoring |
|  | We need a new EASL Guideline on this disease. |
|  | More difussion |
|  | - What is the best marker to evaluate treatment efficacy?  - How to deal with patients with probable pathogen mutations, without signs for an overload of copper storage?  - More research regarding to combination of chelator and zinc therapy |
|  | Value of REC, safety and efficacy of ammonium tetrathiomolybdate |
|  | Diagnosis: genetic test; new exams - PET, radioactive copper, REC; Treatment: first line; monitoring |
|  | Updating EASL 2012 guideline |
|  | availability of drugs in different countries as primary therapy or secondary |
|  | pregnancy/ breastfeeding, ultrasound and elastography data |

*Quoted replies from the 15 respondents answering this final optional question.*

**S4: Supplementary figure “Word cloud from supplementary table 3 – edited for clarity”.
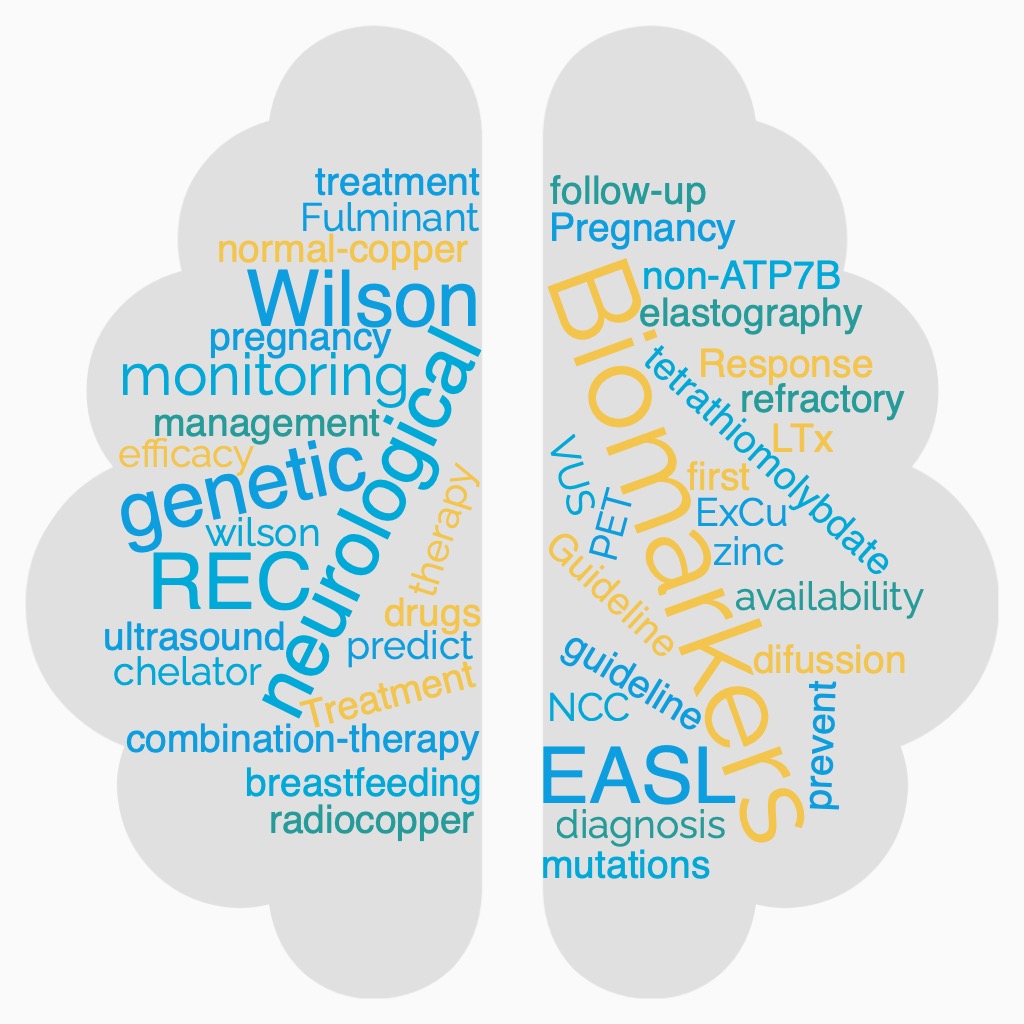
**

Created using https://www.wordclouds.com with a free use policy.
